# Patterns in Habit Formation of New Practices within an Anesthesia Department

**Published:** 2025-03-09

**Authors:** Cindy B Yeoh, Rahul Mhaskar, Sephalie Patel, Jonathan B Cohen, Osama Hafez

**Affiliations:** 1 Department of Anesthesiology, Moffitt Cancer Center, USA; 2 Department of Medical Education, University of South Florida, USA

## INTRODUCTION

Adopting and maintaining new practices is a perennial struggle within healthcare organizations. Habit formation has been identified as a key determinant of behavior change and the challenges of habit formation in the healthcare setting have been accentuated by the COVID-19 pandemic [1]. While numerous studies and best-selling self-help books have examined habit formation at the individual level, the adoption of habits at the group level in the work environment is significantly more complex and studies are sparse. Factors affecting habit formation in the workplace include complexity of desired practice/behavior, associated rewards, workplace environment, and varying habit-formation timetables of different individuals.

As defined in Psychology, habits are automatically triggered responses to associated contextual cues. Habits have been studied and characterized in terms of cognitive, motivational, and neurobiological properties [2]. They have been described as interfacing with goal achievement in various ways; most commonly, they are formed through the deliberate pursuit of goals by repeating the same responses in a given context [3]. According to the literature, habit formation takes an average of 59–70 days [4]. However, it has been suggested that it can take 18–254 days for a person to form a new habit, with the broad time frame attributed to individual differences and complexity of habits considered [5].

This study examines the adoption and retention of practices (i.e., habit formation) in the healthcare environment, to provide guidance for organizations in implementing Quality Improvement (QI) initiatives. By implementing new quality metric goals within the Department of Anesthesiology, we sought to determine the time needed for adopting new QI practices, assess compliance, improvement, sustainability, and differences in adoption speed of implemented QI practices within the department.

## METHODS

Two new QI practices were introduced within the Department of Anesthesiology. Compliance with these newly introduced practice changes was deemed a requirement. QI practices implemented were documentation requirements for smoking and Postoperative Nausea and Vomiting (PONV) quality metrics as set forth by Centers for Medicare and Medicaid Services (CMS) Merit-Based Incentive Payment System (MIPS) program. These practice requirements were introduced sequentially and two years apart.

ComplianceratesfortwodifferentQIpracticesforeachanesthesiologist were compiled and de-identified to ensure confidentiality. Data were then analyzed to determine the time of earliest and latest adoption of QI practices and the time difference between the two. Compliance data for both QI practices were also studied to determine degradation/ sustainability over time and the effect of introducing a second QI practice on performance with the first QI practice.

## RESULTS

**Figure 1:**
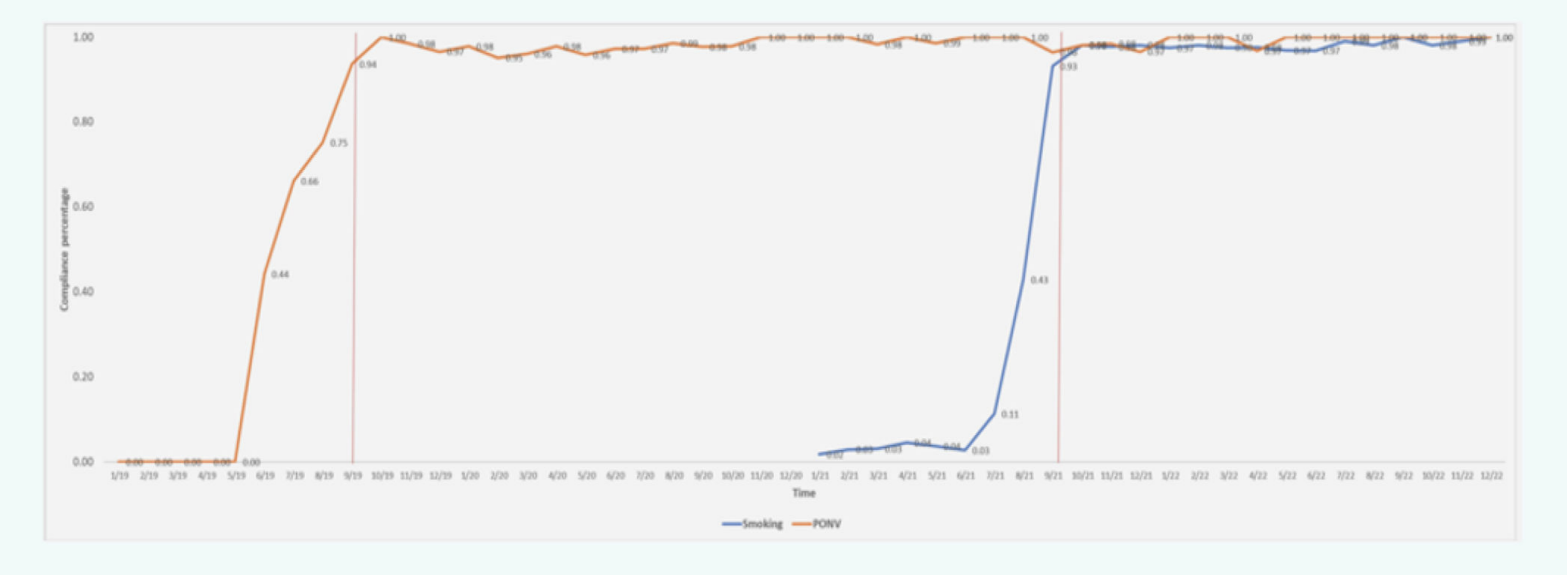
compliance over time. X axis denotes the time and y axis denotes the compliance in percentage. The red vertical lines indicate the time at which the compliance reached the 90% threshold.

We collected data from 19 providers for the PONV-related compliance, and 18 providers contributed to the smoking-related compliance. The median compliance for PONV ranged from 0% to 100%. The median compliance for smoking ranged from 2% to 100%. Providers took eight months from the introduction of the new practice and the target threshold of 90% for habit formation for both PONV and smoking-related quality metrics. Following these eight months, adherence to the QI practice did not drop below the target threshold of 90% for compliance. In fact, after reaching the target threshold, the median compliance was 99% (93% to 100%) and 98% (93% to 100%) for PONV and smoking, respectively. The introduction of a second QI practice (2 years later) did not cause a drop in adherence to the first practice (Figure 1).

Consistent with models on individual habit formation, our results demonstrate that group habits are acquired and established after a period of incremental strengthening following an asymptotic curve. The relationship between behavior repetition and habit automaticity is not linear [5]. According to Bargh et al., factors determining habit automaticity include efficiency, lack of awareness, intention, and control of behavior [6]. The plateau of the asymptotic curve represents peak habit automaticity. Whether a behavior is established as habit early on with large increases, or after a longer period with numerous repetitions, additional contextual repetitions no longer increase automaticity. Our findings also support persistence and sustainability of habits formed over time once automaticity is achieved.

## DISCUSSION

The pressure to readily adopt new QI practices in healthcare is ever-present. This study sheds light on important questions like duration to achieve and sustain practice change through habit formation and optimal timing for introducing new practices. Further research in the realm of group habit formation can guide department leadership in determining the best plan for rolling out QI practices, optimizing practice change, and maximizing practice adoption.
